# The Sec11 C-terminal short helix promotes productive engagement of internal signal sequences with the signal peptidase complex

**DOI:** 10.1016/j.jbc.2026.113442

**Published:** 2026-08-12

**Authors:** Yeonji Chung, Mariia Borbuliak, Sanghun Hwang, Paulo C.T. Souza, Hyun Kim

**Affiliations:** 1 School of Biological Sciences and Institute of Biodiversity, Seoul National University, Seoul, South Korea; 2 Laboratoire de Biologie et Modélisation de la Cellule, UMR 5086, CNRS, Ecole Normale Supérieure de Lyon, Lyon, France; 3 Centre Blaise Pascal de Simulation et de Modélisation Numérique, Ecole Normale Supérieure de Lyon, Lyon, France

**Keywords:** Sec11, signal peptidase, intracellular processing, signal sequence, signal peptide, substrate specificity, yeast, endoplasmic reticulum (ER)

## Abstract

Proteins destined for secretion typically contain N-terminal signal sequences that target nascent chains to the endoplasmic reticulum. Following targeting, these sequences are cleaved by the heterotetrameric signal peptidase complex (SPC). Despite a conserved N-terminal, hydrophobic core, and C-terminal tripartite organization, their sequences are highly variable. How SPC subunits contribute to recognition of these diverse sequences remains poorly understood. Sec11, the catalytic subunit of the SPC, contains an N-terminal transmembrane domain and a C-terminal hydrophobic region. The latter was unresolved in human SPC cryo-EM structures, likely due to intrinsic flexibility, yet AlphaFold predictions of the yeast SPC suggest that this region forms a C-terminal short helix (CTS) strategically positioned within the presumed signal sequence-binding site. This structural arrangement led us to hypothesize its possible role in substrate handling during signal peptide processing and we undertook to investigate its function using biochemical assays combined with molecular dynamics simulations. Topology mapping confirmed that the Sec11 CTS traverses the endoplasmic reticulum membrane and molecular dynamics simulations showed that removal of the Sec11 CTS does not substantially alter overall SPC architecture or the proximal membrane environment. While N-terminal signal sequences of varying hydrophobicity were efficiently cleaved, internal signal sequences with extended N-terminal region were selectively defective in cleavage in the absence of the Sec11 CTS. These data suggest that the Sec11 CTS specifically stabilizes internal signal sequences for productive engagement with SPC.

In eukaryotic cells, approximately 30% of the proteome is destined for secretion or for localization in subcellular organelles of the secretory pathway. These proteins typically contain signal sequences at their N-termini that facilitate three distinct stages of protein translocation across the endoplasmic reticulum (ER) (1, 2, 3, 4). First, these sequences target nascent chains to the ER membrane by interacting with the signal recognition particle (SRP), which delivers the ribosome–nascent chain complex to the Sec61 translocon. Upon targeting, signal sequences trigger the opening of the Sec61 protein–conducting channel by binding at its lateral gate. Subsequently, they ensure proper folding and localization of nascent chains through timely processing by the signal peptidase complex (SPC).

Signal sequences share a conserved tripartite structure consisting of an N-terminal region (n-region) enriched with basic residues, a hydrophobic helical core (h-region), and a polar C-terminal region (c-region) containing the cleavage site (2, 5). Despite this simple organization, signal sequences are highly diverse in amino acid composition and in the length of each region (6, 7). Therefore, some signal sequences are cleaved by SPC, becoming signal peptides, whereas others are uncleaved, becoming signal anchors in the membrane.

The SPC is a heterotetrameric membrane complex. Cryo-EM structure of the human SPC reveals that Sec11 and Spc3 form the luminal catalytic core, whereas Spc1 and Spc2 reside predominantly on the cytosolic side of the ER membrane (8). All four subunits contact through their transmembrane (TM) domains. A unique structural feature of the SPC is the formation of 2 TM helical bundles, Sec11 with Spc2 and Spc3 with Spc1, which create a gap between them referred to as the TM window (8). Molecular dynamics (MD) simulations showed that the membrane within the TM window is thinner than the surrounding lipid bilayer (8), suggesting a potential mechanism for substrate selection based on h-region length. Signal peptides, which typically possess shorter h-region, could be accommodated in this thinner membrane environment and cleaved, whereas signal anchored sequences with longer h-region are excluded, thus remain uncleaved. Consistent with this model, removal of Spc2 subunit which altered TM window thickness disrupted proper discrimination between signal peptides and signal anchored sequences (9).

However, h-region length is only 1 variable among many in signal sequences. Particularly, length of the n-region (from the N terminus to the start of the h-region) is the most varying feature (2, 6), and depending on which, signal sequences can be classified as either N-terminal or internal signal sequences. How these distinct classes are differentially recognized by the SPC remains unclear.

Sec11, the catalytic subunit of the SPC, is evolutionarily conserved from bacteria to humans (10, 11). It contains an N-terminal TM domain and the C-terminal hydrophobic region. The latter was unresolved in the human SPC cryo-EM structure, likely due to intrinsic flexibility (8). However, AlphaFold predictions of the yeast SPC suggest that this C-terminal hydrophobic region forms a short helix positioned within the TM window, the presumed signal sequence-binding site (8). Guided by this structural feature, we hypothesized that the Sec11 C-terminal short helix (CTS) contributes to substrate recognition and/or stabilization during processing. To test this, we examined the membrane topology of the CTS, the substrate specificity of SPC lacking the Sec11 CTS, and the dynamic behaviors of SPC with and without the Sec11 CTS using biochemical assays and MD simulations.

MD simulations revealed that deletion of the Sec11 CTS does not markedly alter the overall SPC architecture or the membrane properties of the TM window. Consistently, SPC cleavage assays showed efficient processing of N-terminal signal sequences of varying hydrophobicity. In contrast, SPC lacking the Sec11 CTS exhibited a selective defect in processing internal signal sequences. Furthermore, MD simulations indicate reduced stability of the internal signal sequence binding, particularly for substrates with extended n-region in the absence of Sec11 CTS. These findings suggest that, rather than modulating SPC structure or the proximal membrane environment, the Sec11 CTS stabilizes internal signal sequences to position properly in the TM window, thereby promoting their cleavage by SPC.

## Results

### Membrane orientation of the Sec11 CTS

**Figure 1:**
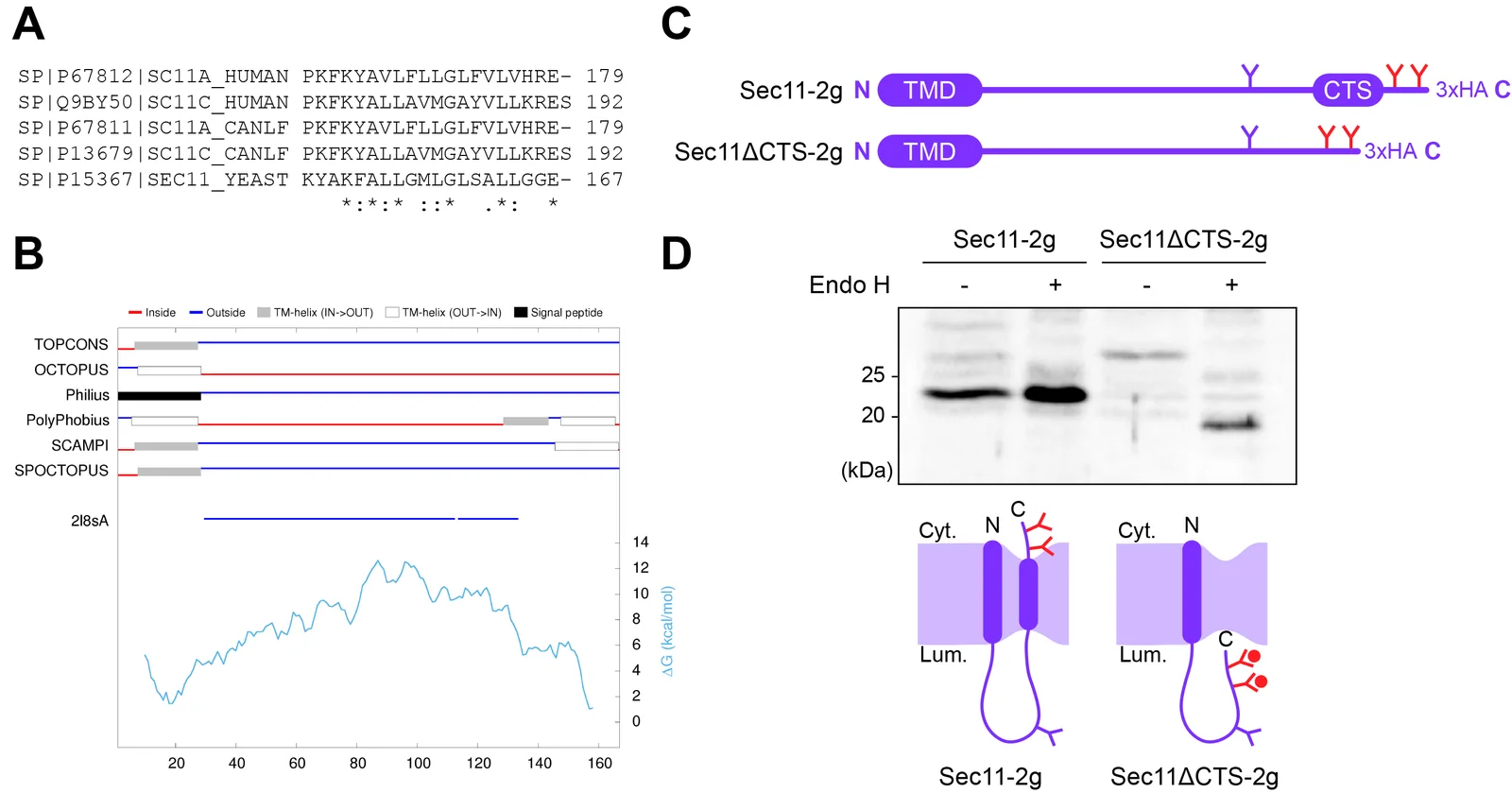
**Membrane topology of the C-terminal short helix (CTS) of Sec11.***A*, alignment of the C-terminal 20 residues of human, canine Sec11A and yeast Sec11, and 21 residues of human and canine Sec11C (human: *Homo sapiens*, canine (CANLF): *Canis lupus familiaris*, yeast: *S. cerevisiae*). *B*, membrane topology predictions of yeast Sec11 generated by TOPCONS (39). *C*, schematic representation of the Sec11-2g and Sec11ΔCTS-2g constructs. TMD, transmembrane domain; CTS, C-terminal short helix; 3XHA, triple hemagglutinin; Y, glycosylation site (engineered sites are in *red*). *D*, W303-1α cells expressing Sec11-2g or Sec11ΔCTS-2g were analyzed by SDS-PAGE and Western blotting. Schematic models of the membrane topology of Sec11-2g and Sec11ΔCTS-2g are shown below. *Filled circles* in glycosylation sites indicate glycosylation.

Sec11 is the catalytic subunit of the SPC and contains an N-terminal TM domain as well as a weakly hydrophobic short C-terminal segment that is not predicted as a TM by most membrane protein prediction algorithms (Fig. 1, *A* and *B*). We therefore sought to determine the membrane topology of this segment, hereafter referred to as the CTS.

To assess CTS orientation, we introduced two N-linked glycosylation sites into the linker region between Sec11 and a C-terminal triple hemagglutinin tag (3XHA) in both full-length Sec11 and CTS-truncated Sec11 (Sec11ΔCTS), generating Sec11-2g and Sec11ΔCTS-2g constructs, respectively (Fig. 1*C*). Although Sec11 contains an endogenous luminal N-linked glycosylation site, it did not bind Con A, indicating that this site was unglycosylated (12). If the CTS forms a TM helix, the C terminus of full-length Sec11 would be oriented toward the cytosol, preventing glycosylation of Sec11–2g, whereas deletion of the CTS would place the C terminus in the ER lumen, allowing Sec11ΔCTS–2g to be glycosylated.

Consistent with this model, only Sec11ΔCTS-2g was sensitive to endoglycosidase H (Endo H) treatment, whereas Sec11-2g remained Endo H–resistant (Fig. 1*D*). These results indicate that the C terminus of Sec11 faces the cytosol and that the CTS spans the ER membrane.

### Growth phenotype of yeast cells lacking the Sec11 CTS

Having established that the Sec11 CTS traverses the ER membrane, we next examined the physiological consequences of CTS deletion using genetic complementation and growth assays.

To determine whether the Sec11 CTS is required for Sec11 function *in vivo*, we performed a genetic complementation assay by plasmid shuffling in *Saccharomyces cerevisiae* (13). Because *SEC11* encodes the sole catalytic subunit of the SPC, it is essential for yeast viability. The chromosomal copy of *SEC11* was deleted in the presence of a *URA*-borne plasmid expressing WT *SEC11*. In parallel, plasmids expressing CTS-truncated *sec11* (*sec11*ΔCTS) or C terminally GFP-tagged *SEC11* (*SEC11-GFP*) were constructed and introduced into the *sec11*Δ strain.

The Sec11 CTS was unresolved in the human SPC cryo-EM structure, suggesting intrinsic flexibility (8). Given that the Sec11 CTS is weakly hydrophobic and predicted to be either soluble or a TM segment (Fig. 1*B*), we hypothesized that it may dynamically associate with the membrane. If so, C-terminal GFP tagging could potentially interfere with its function *in vivo*.

Plasmid shuffling was then performed by counterselection on medium containing 5-fluoroorotic acid (5-FOA). In this assay, functional Sec11 variants replace the *URA*-borne WT *SEC11* plasmid, allowing growth on 5-FOA, whereas nonfunctional variants fail to support viability, resulting in cell death due to conversion of 5-FOA to toxic 5-fluorouracil.

**Figure 2:**
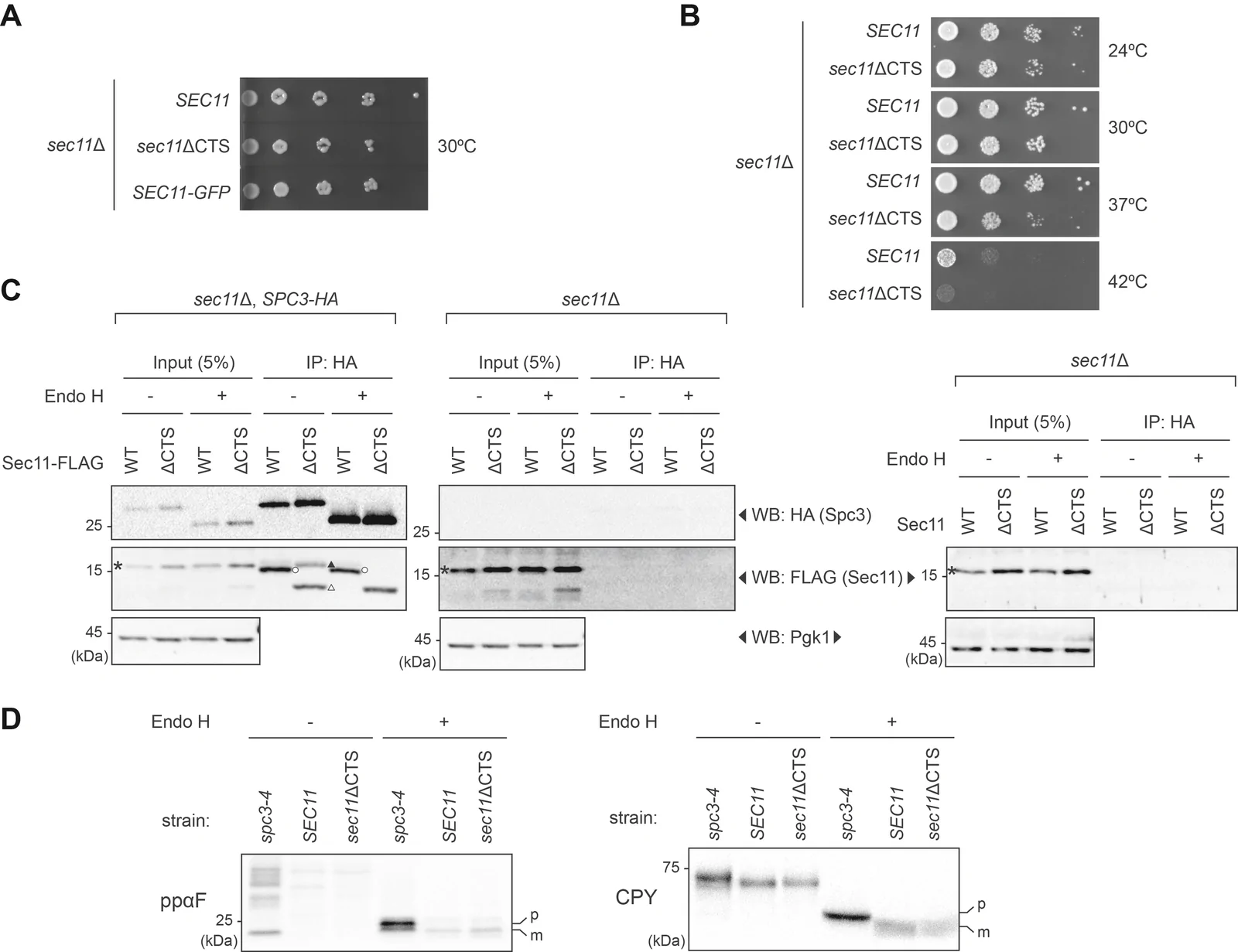
**Characterization of the Sec11 CTS deletion.***A*, growth assays of *sec11*Δ cells bearing plasmids encoding *SEC11*, *sec11*ΔCTS, or *SEC11-GFP* under the control of endogenous promoter at 30 °C. *B*, growth assays of *sec11*Δ cells bearing plasmids encoding *SEC11* or *sec11*ΔCTS under the control of endogenous promoter at the indicated temperatures. Cells (0.1 OD_600_ unit) were serially diluted by 10-fold, spotted onto –Leu plates and incubated for 36 h. *C*, coimmunoprecipitation experiments. Crude membranes were prepared from the cells expressing FLAG-tagged Sec11 or Sec11ΔCTS together with HA-tagged Spc3 (*left*), FLAG-tagged Sec11 or Sec11ΔCTS with untagged Spc3 (*middle*), or untagged Sec11 or Sec11ΔCTS with untagged Spc3 (*right*). 5% of the whole-cell lysate was loaded as “input (5%)”. *Open circles* (○) indicate unglycosylated Sec11; *filled* (▲) and *open* (△) *triangles* indicate glycosylated and unglycosylated Sec11ΔCTS, respectively. *Asterisks* (∗) indicate nonspecific band. *D*, 5 min pulse labeling of prepro-α-factor (ppαF, *left*) and CPY (*right*) in the *spc3-4*, *sec11*Δ+*SEC11*, and *sec11*Δ+*sec11*Δ*CTS* strains with [^35^S]Met. P, precursor; m, mature form; CTS, C-terminal short helix; CPY, carboxypeptidase Y.

Both Sec11ΔCTS and GFP-tagged Sec11 supported growth on 5-FOA, indicating that each variant can functionally replace WT Sec11 (Fig. 2*A*). However, cells expressing Sec11ΔCTS exhibited slower growth than cells expressing WT or GFP-tagged Sec11. To quantitatively assess this growth defect, serial dilutions of yeast cells were spotted onto solid medium and incubated for 36 h at 24 °C, 30 °C, 37 °C, or 42 °C (Fig. 2*B*). While Sec11ΔCTS cells grew comparably to the WT cells at lower temperatures, they displayed a pronounced growth defect at elevated temperatures (37 °C and 42 °C). These results indicate that although the Sec11 CTS is not essential for viability under standard conditions, it becomes important for optimal cell growth at higher temperatures, suggesting a role in maintaining SPC function under thermal stress.

### Stability and catalytic function of SPC lacking the Sec11 CTS

Next, we assessed whether deletion of the Sec11 CTS affects the stability or catalytic activity of the SPC. To examine whether interactions between the catalytic core subunits Sec11 and Spc3 are maintained in the absence of the Sec11 CTS, we performed co-immunoprecipitation (co-IP) experiments. *SPC3* was chromosomally tagged with triple HA, and *SEC11* was deleted with plasmids expressing either FLAG-tagged WT *SEC11* or *sec11*ΔCTS under the control of the endogenous promoter. Crude membranes were prepared, Spc3-HA was immunoprecipitated using anti-HA antibodies, and associated Sec11 proteins were detected by immunoblotting with anti-FLAG antibodies. We focused on these two subunits because SPC complexes without Spc1 and Spc2 retain catalytic activity (14).

Both Sec11 and Sec11ΔCTS were coimmunoprecipitated with Spc3 (Fig. 2*C*, left panel), indicating that Sec11 associates with Spc3 regardless of the presence of the CTS. Because Sec11 primarily interacts with Spc3 through their luminal domains (8), these results suggest that the Sec11 CTS is not required for assembly of the catalytic core. We noted a weakly glycosylated form of Sec11ΔCTS, whereas WT Sec11 appeared predominantly in an unglycosylated form. This partial glycosylation of Sec11ΔCTS likely reflects increased accessibility of an otherwise inefficient endogenous N-linked glycosylation site following CTS deletion.

As controls, co-IP was performed with *sec11*Δ cells expressing FLAG-tagged *SEC11* or *sec11*ΔCTS in the absence of HA tagging on *SPC3*, confirming the specificity of the interaction (Fig. 2*C*, middle panel). Bands in input samples were also detected in lysates of cells expressing untagged Sec11 variants (Fig. 2*C*, right panel), indicating nonspecific proteins cross-react with the anti-FLAG antibody. Together, these data demonstrate that truncation of the Sec11 CTS does not disrupt interaction between the catalytic core subunits.

To evaluate whether loss of the Sec11 CTS affects SPC catalytic activity, we examined processing of two endogenous secretory proteins, prepro–α factor (ppαF) and carboxypeptidase Y (CPY), by 5 min pulse-labeling of *sec11*Δ cells expressing either WT *SEC11* or *sec11*ΔCTS with [^35^S]Met. As a control for impaired SPC activity, these substrates were also analyzed in the temperature sensitive *spc3-4* mutant, which accumulates uncleaved precursors at the nonpermissive temperature of 37 °C (10).

In contrast to *spc3-4* cells, both ppαF and CPY were fully processed in *sec11*Δ cells expressing either WT *SEC11* or *sec11*ΔCTS during a 5 min pulse (Fig. 2*D*). These results indicate that deletion of the Sec11 CTS does not impair SPC-mediated cleavage of secretory precursors. Collectively, our data suggest that truncation of the Sec11 CTS neither destabilizes the SPC catalytic core nor compromises its proteolytic activity.

### SPC lacking the Sec11 CTS efficiently cleaves N-terminal signal sequences of varying hydrophobicity

**Figure 3:**
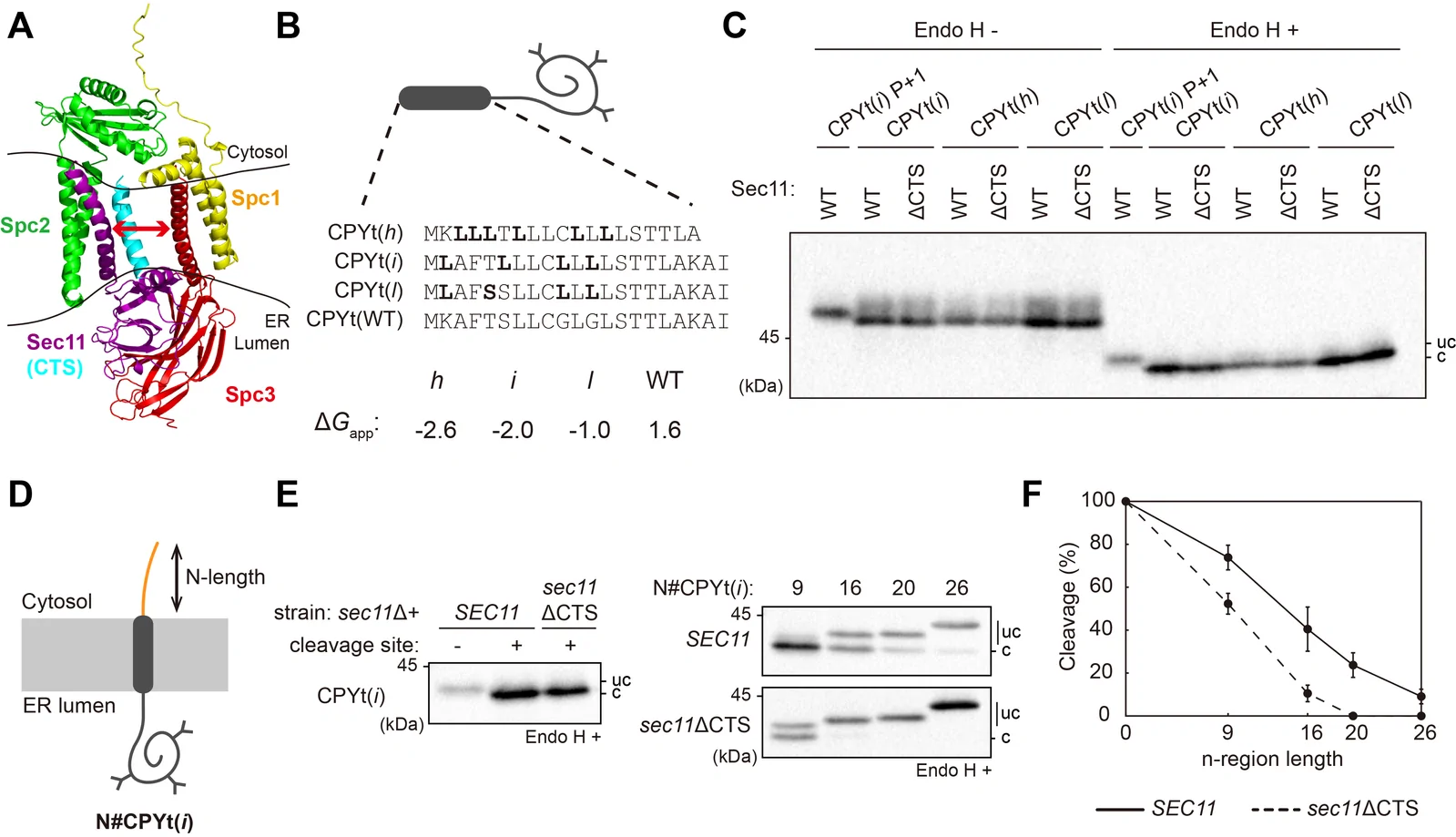
**Cleavage efficiencies of signal sequences with varying n-region length.***A*, AlphaFold2-predicted structure of the yeast SPC with Spc1 in *yellow*, Spc2 in *green*, Spc3 in *red*, Sec11 in *purple*, and the Sec11 CTS in *cyan*. The TM window of SPC is indicated with a *red arrow*. *B*, signal sequences and hydrophobicities of CPY and its variants. *h*, high; *i*, intermediate; *l*, low. Hydrophobicity is shown as Δ*G*_app_ (kcal/mol) (40) *C*, CPY variants with varying hydrophobicity. Samples were analyzed by Western blotting as in Figure 1*D*. CPYt(*i*) P + 1, cleavage site mutant. *D*, schematic representation of N#CPYt(*i*) variants with different n-region length. *E*, Western blot analysis of N-terminal CPYt(*i*) together with its cleavage-site mutant (*left*) and CPYt(*i*) variants with n-region lengths of 9 to 26 (N#CPYt(*i*)). *F*, cleavage efficiencies from (E) were quantified and plotted. Mean values from three independent experiments are shown with SDs. uc, uncleaved; c, cleaved. CTS, C-terminal short helix; CPY, carboxypeptidase Y; n-region, N-terminal region; SPC, signal peptidase complex; TM, transmembrane.

Consistent with our topology mapping (Fig. 1*D*), AlphaFold predicted yeast SPC model suggests that the Sec11 CTS spans the membrane and positions within the TM window where the signal sequence h-region resides (Fig. 3*A*) (8). This structural arrangement led us to hypothesize that the Sec11 CTS might contribute to signal sequence recognition based on h-region hydrophobicity.

To test whether loss of the Sec11 CTS affects SPC-mediated cleavage of signal sequences with different hydrophobicities, we analyzed processing of CPY variants containing signal sequences of low (l), intermediate (i), or high (h) hydrophobicity (Fig. 3*B*). All CPY variants were glycosylated, confirming efficient ER targeting. A CPYt(i) construct carrying a cleavage site mutation (CPYt(i) P + 1) was included as a size reference. Western blotting analysis showed efficient cleavage of all three CPY variants in both WT and *sec11*ΔCTS cells (Fig. 3*C*), indicating that deletion of the Sec11 CTS does not impair SPC-mediated processing of N-terminal signal sequences across a broad range of hydrophobicity.

In *S. cerevisiae*, secretory proteins are targeted to the ER *via* either SRP-dependent cotranslational or Sec62/Sec63-dependent posttranslational translocation pathways, depending largely on signal sequence hydrophobicity (14, 15, 16). Previously, we showed that CPY is translocated by the Sec62/Sec63-dependent pathway, whereas more hydrophobic CPY variants are translocated by the SRP-dependent pathway (17). Therefore, these results imply that SPC lacking the Sec11 CTS remains competent to cleave signal sequences regardless of translocation pathways.

### Cleavage of internal signal sequences is impaired in the absence of the Sec11 CTS

Among the three regions defining signal sequence architecture (n-, h-, and c-regions), the n-region exhibits the greatest variability, ranging from approximately 3 to over 100 amino acids in length (2, 6). How the SPC accommodates signal sequences with such diverse n-region lengths remains poorly understood. Previously, we showed that cleavage efficiency decreases as n-region length increases, suggesting that the SPC processes internal signal sequences less efficiently than N-terminal signal sequences (18).

To determine whether the Sec11 CTS contributes to substrate selection based on n-region length, we examined cleavage of a series of N#CPYt(i) constructs containing progressively longer n-regions in *sec11*Δ cells expressing either WT *SEC11* or *sec11*ΔCTS (Fig. 3*D*). Substrates with short n-regions were efficiently cleaved in both strains (Fig. 3*E*). In contrast, cleavage of substrates with longer n-regions was reduced by approximately 20% in *sec11*ΔCTS cells relative to WT cells (Fig. 3*F*). These results indicate that SPC lacking the Sec11 CTS is selectively impaired in processing internal signal sequences.

**Figure 4:**
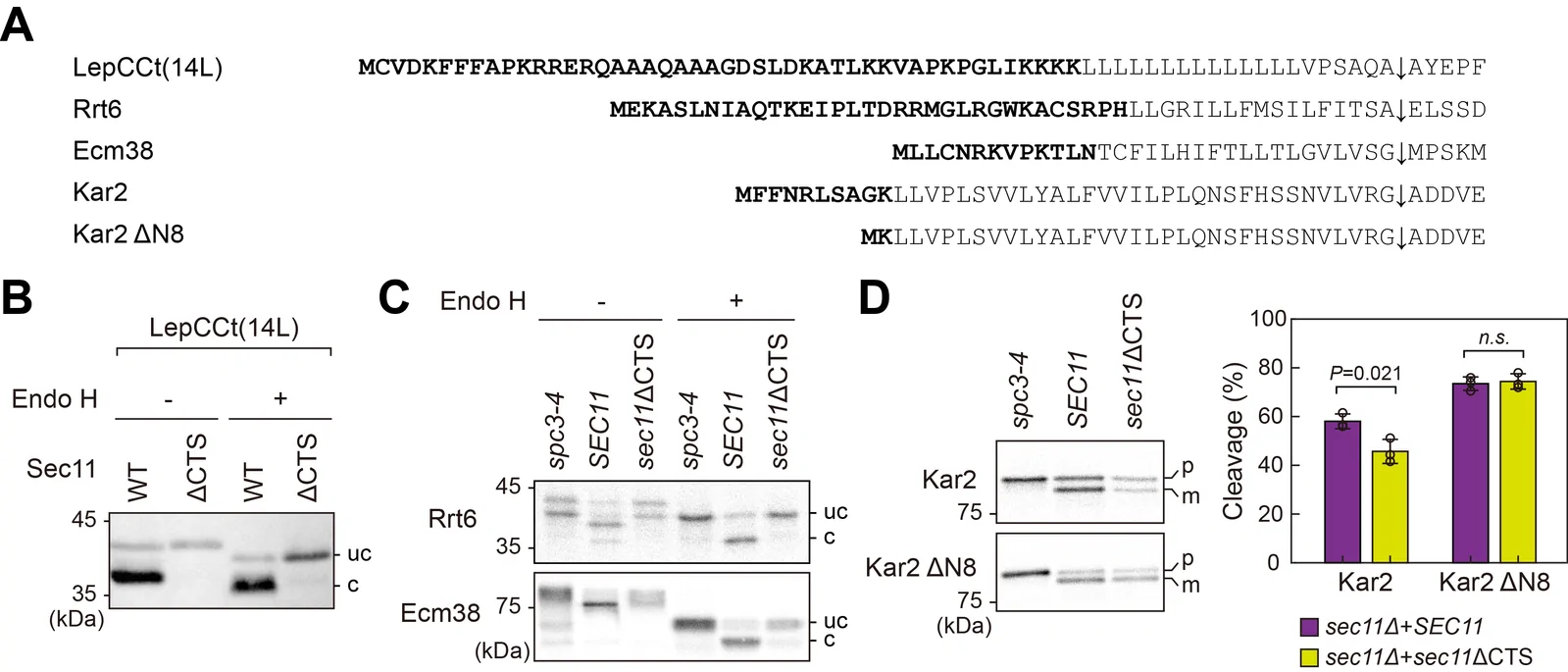
**Cleavage efficiencies of internal signal sequences.***A*, internal signal sequences of LepCCt(14L), Rrt6, Ecm38, Kar2, and N terminally truncated Kar2 (Kar2ΔN8). N regions are indicated in *bold*. *Downward arrows* indicate predicted cleavage sites identified by SignalP 6.0 (41). *B*, Western blot analysis of LepCCt(14L). *C*, a 5-min pulse labeling of *spc3-4*, *sec11*Δ+*SEC11*, and *sec11*Δ+*sec11*ΔCTS cells expressing Rrt6 (*top*) and Ecm38 (*bottom*) with [^35^S]Met. *D*, a 5-min pulse labeling of *spc3-4*, *sec11*Δ+*SEC11*, and *sec11*Δ+*sec11*ΔCTS cells expressing Kar2 or Kar2ΔN8. Average cleavage efficiencies from three independent experiments are shown with SDs. Statistical analyses were performed using an unpaired Student’s *t* test (*n.s*., not significant). c, cleaved; CTS, C-terminal short helix; m, mature forms; P, precursor; uc, uncleaved.

To further validate this finding, we analyzed additional model and endogenous substrates containing internal signal sequences. The model substrate LepCCt(14L) contains a 46–amino acid n-region (18, 19), whereas the endogenous proteins Rrt6, Ecm38, and Kar2 possess n-regions of 33, 13, and 10 amino acids, respectively (Fig. 4*A*). These constructs were expressed in *sec11*Δ cells carrying plasmids encoding either WT *SEC11* or *sec11*ΔCTS, and SPC-mediated cleavage was assessed.

Cleavage of LepCCt(14L) was markedly reduced in cells lacking the Sec11 CTS (Fig. 4*B*). For Rrt6, lysates from both *spc3-4* and *sec11*ΔCTS cells contained mixtures of unglycosylated and glycosylated forms, suggesting partial targeting defects (Fig. 4*C*). Among the glycosylated, ER-targeted proteins, we observed efficient SPC-mediated cleavage in WT cells, whereas the majority of Rrt6 remained uncleaved in *sec11*ΔCTS cells (Fig. 4*C*). Similarly, cleavage of Ecm38 was also diminished in *sec11*ΔCTS cells compared with WT cells (Fig. 4*C*).

Consistently, processing of Kar2 decreased from 58% in WT cells to 45.7% in *sec11*ΔCTS cells (Fig. 4*D*). Notably, deletion of the N-terminal eight residues of the Kar2 signal sequence (Kar2ΔN8) restored cleavage efficiency in *sec11*ΔCTS cells to levels comparable to WT cells (Fig. 4*D*), indicating that shortening the n-region reduces the Sec11 CTS dependency.

Together, these results demonstrate that the Sec11 CTS is specifically required for efficient cleavage of internal signal sequences, particularly those with extended n-regions, while cleavage of N-terminal signal sequences remains unaffected.

### Cleavage of pre-β-lactamase in yeast cells

**Figure 5:**
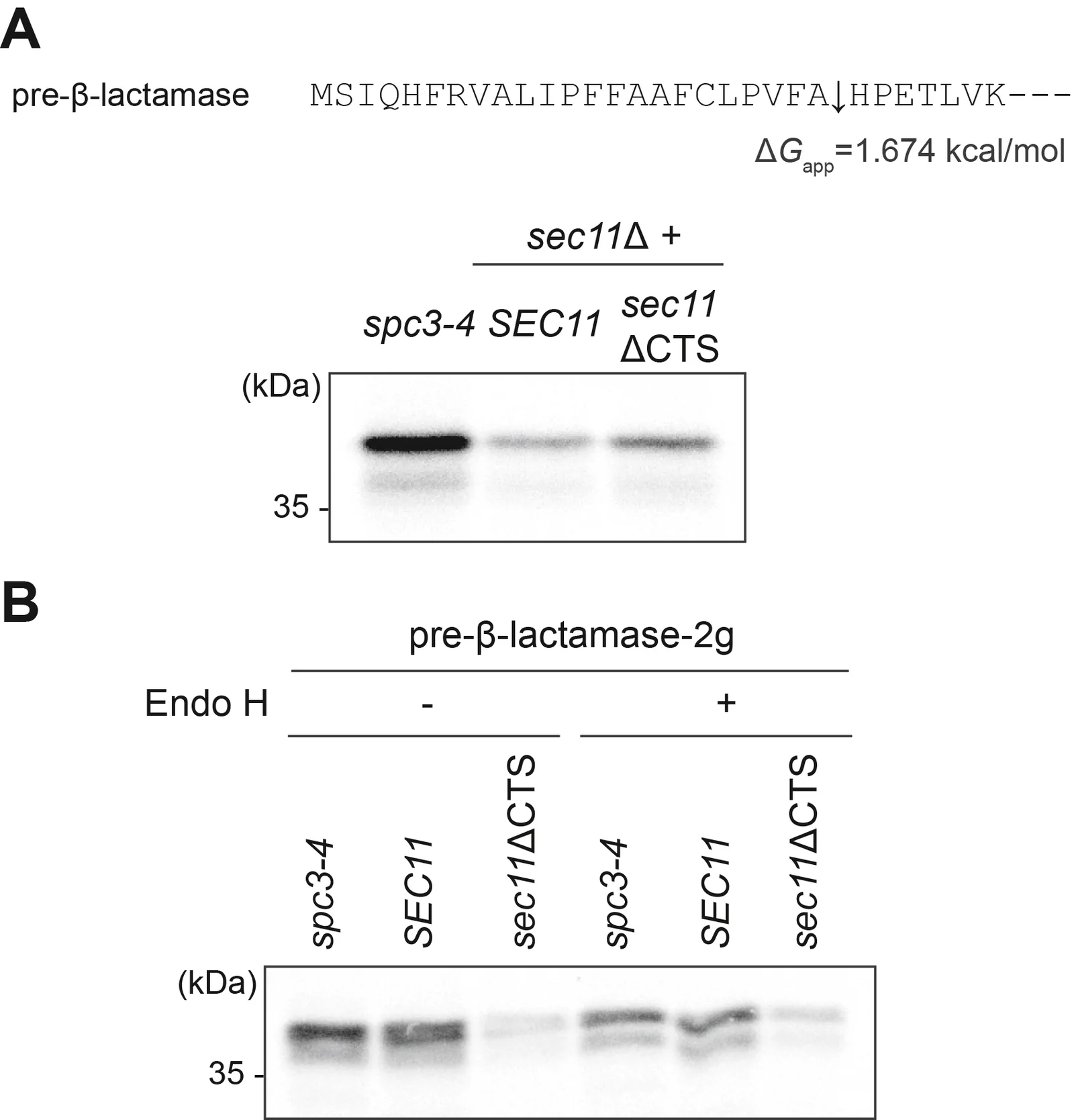
**Targeting of *E. coli* pre-β-lactamase.***A*, N-terminal protein sequence of *E. coli* pre-β-lactamase (*top*). Hydrophobicity is shown as Δ*G*_app_ (kcal/mol) (40). A *downward arrow* indicates the predicted signal sequence cleavage site. A 5-min pulse labeling of *spc3-4*, *sec11*Δ+*SEC11*, and *sec11*Δ+*sec11*ΔCTS cells expressing pre-β-lactamase (*bottom*) with [^35^S]Met. *B*, a 5-min pulse labeling of *spc3-4*, *sec11*Δ+*SEC11*, and *sec11*Δ+*sec11*ΔCTS cells expressing pre-β-lactamase carrying N-linked glycosylation sites at the C terminus with [^35^S]Met. CTS, C-terminal short helix.

Previous work showed that signal sequence cleavage of *in vitro* translated *Escherichia coli* pre-β-lactamase is impaired in purified human SPC complexes lacking the Sec11 CTS (8). To determine whether cleavage of pre-β-lactamase is similarly affected in yeast cells lacking the Sec11 CTS, we analyzed processing of pre-β-lactamase in *sec11*ΔCTS, WT, and *spc3-4* cells by 5 min pulse labeling. The migration pattern of pre-β-lactamase in *sec11*ΔCTS cells was indistinguishable from that observed in *spc3-4* cells, indicating that the protein remained uncleaved (Fig. 5*A*). Unexpectedly, pre-β-lactamase was also not processed in WT cells (Fig. 5*A*).

Because it was unclear whether this lack of processing reflected defective ER targeting or impaired SPC-mediated cleavage, we introduced two N-linked glycosylation sites at the C terminus of pre-β-lactamase (pre-β-lactamase-2g) to monitor ER translocation (Fig. 5*B*). Endo H treatment revealed that pre-β-lactamase-2g was Endo H resistant, indicating that it failed to enter the yeast ER (Fig. 5*B*).

The signal sequence of pre-β-lactamase exhibits low hydrophobicity (Δ*G*_app_ of 1.67 kcal/mol), similar to CPY signal sequence, which utilizes the SRP-independent posttranslational pathway in yeast (20). Previous study has shown that *E .coli* β-lactamase adopts a native-like conformation in the cytoplasm prior to translocation when expressed in *S. cerevisiae* (21). We therefore speculate that *E. coli* pre-β-lactamase folds during posttranslational targeting, preventing its translocation across the ER membrane. As a consequence, the protein does not encounter the SPC, explaining the absence of signal peptide cleavage in yeast cells.

### MD simulations of the AlphaFold predicted yeast SPC

Previous studies demonstrated membrane thinning at the TM window in both human and yeast SPC (8, 9). The thickness at the TM window also changed in the absence of Spc2 (9). To assess how membrane properties change in the presence of Sec11 WT and ΔCTS, we performed coarse-grained (CG) MD simulations of the yeast SPC AlphaFold model, which was previously generated and validated (9). The protein model with WT SPC or SPC lacking the Sec11 CTS was embedded in a complex lipid mixture that mimics the ER lipid environment using Martini models for proteins and the Martini 3 lipidome models (22, 23, 24).

**Figure 6:**
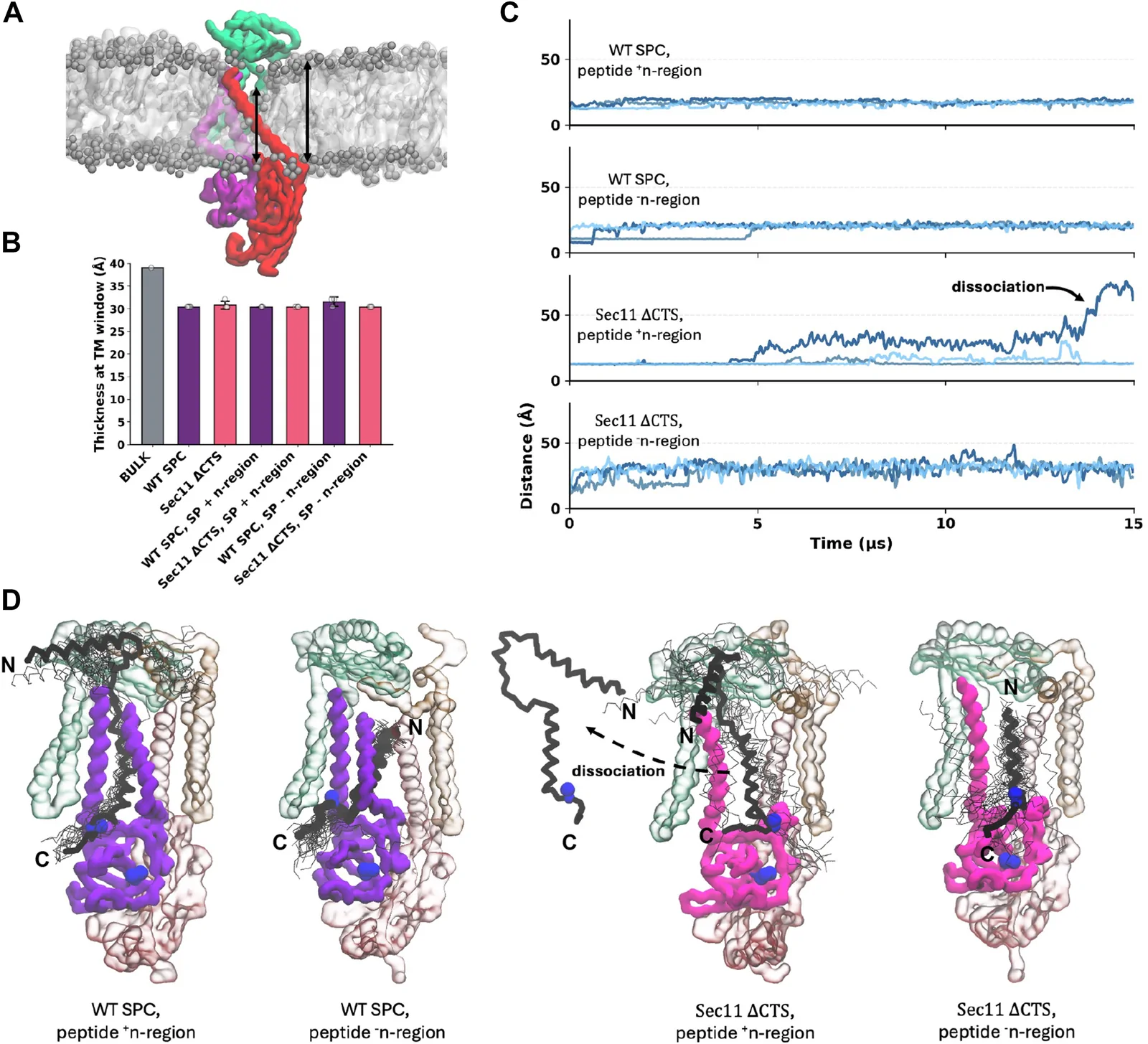
**Molecular dynamics simulations of yeast SPC with Sec11 WT and ΔCTS.***A*, MD simulation snapshot of SPC in ER lipid mixture demonstrating membrane thinning at the TM window of SPC. Spc2 is shown in *green*, Spc3 in *red*, Sec11 in *purple*, and Spc1 is not shown for clarity. *Top* is the cytoplasmic and *bottom* is the lumen sides of the ER membrane. The phosphate head groups (Martini PO_4_ beads) are colored in *gray*, and the lipid tails are represented with a transparent *gray* surface. *B*, comparison of membrane thickness at the TM window of SPC Sec11 WT (*purple*) and ΔCTS (*magenta*), as well as for the systems containing LepCCt(14L) with and without n-region. *C*, comparison of distances between the LepCCt(14L) peptide and SPC Sec11 or ΔCTS. Distances were calculated between the ALA backbone beads of the peptide and HIS83 of Sec11, located at the catalytic site. Each line shows data from 1 simulation repeat per system, colored from *dark to light blue*, with each repeat lasting 15 μs. *D*, representative snapshots of the SPC Sec11 WT (*purple*) and ΔCTS (*magenta*) in the presence of the LepCCt(14L) peptide with and without n-region. The SPC subunits are represented with a transparent surface colored as in (*A*), with Spc1 shown in *orange*. The ALA and HIS83 residues of the peptide and Sec11, respectively, used for distance calculations, are shown as *blue spheres*. The representative position of LepCCt(14L) peptide is shown in *thick black*, and the other peptide positions during the simulations are shown in *thin black*. CTS, C-terminal short helix; ER, endoplasmic reticulum; MD, molecular dynamics; n-region, N-terminal region; SPC, signal peptidase complex; TM, transmembrane.

As expected, our MD simulations revealed membrane thinning at the TM window in the WT SPC systems, with a thinning of approximately 22% compared to the bulk lipid environment (Fig. 6, *A* and *B*). This is slightly less than previously observed for yeast SPC (9), but considering the new lipid models and differences in the thickness calculation approach, it remains within the expected range. In the absence of Sec11 CTS, membrane thinning at the TM window was similar, with an approximately 21% reduction compared to the bulk membrane (Fig. 6*B*). This suggests that the presence or absence of Sec11 CTS does not substantially affect the degree of membrane thinning at the TM window in the yeast SPC.

As described earlier, the cleavage efficiency of internal signal sequences, such as LepCCt(14L), by the yeast SPC depends on the presence of Sec11 CTS. To understand if the presence of LepCCt(14L) peptide with SPC affects membrane properties, we used AlphaFold predicted models of the yeast SPC with the LepCCt(14L) peptide. Additionally, we assessed how membrane properties near the SPC change if the n-region of the LepCCt(14L) is reduced. The protein models were embedded in the ER lipid mixture described above, and MD simulations were performed. Similarly, as in the SPC with WT Sec11 and ΔCTS, membrane thinning at the TM window was observed in all systems with the tested peptides, with thinning percentages ranging from 19 to 22% (Fig. 6*B*).

Next, we evaluated the change in the position of LepCCt(14L) with and without the n-region relative to the catalytic region at SPC WT Sec11 and ΔCTS. For this, we calculated the distances between the HIS at the catalytic site of Sec11 and ALA (Ala-Leu-Ala) of the peptide adjacent to the cleavage site. In the SPC Sec11 WT systems, both peptides with the full LepCCt(14L) sequence and with a reduced n-region displayed an average position between 1.5 nm and 2 nm from the catalytic residue (Fig. 6, *C* and *D*), consistent with their initial AlphaFold-predicted positions. In the absence of Sec11 CTS, the LepCCt(14L) peptide, with a reduced n-region, also displayed interactions with SPC; however, the peptide slightly shifted its localization within the TM window with an average distance of approximately 2.99 nm. Notably, the full sequence LepCCt(14L) diffused away from the SPC in the SPC ΔCTS in one of the simulated replicas (Fig. 6, *C* and *D*), indicating a higher propensity for dissociation.

To further characterize the interactions between LepCCt(14L) and Sec11, we performed a pairwise contact-frequency analysis using the last 10 μs of each simulation (Fig. S1). In WT SPC, the full-length LepCCt(14L) peptide exhibited contacts between its h-region and the Sec11 CTS, with average contact frequencies reaching approximately 0.6 (Fig. S1*A*, B), identifying the Sec11 CTS as the primary interaction interface within the TM window. The corresponding peptide with a shortened n-region showed higher average contact frequencies (∼1.0) with the same Sec11 CTS (Fig. S1*C*), indicative of a more stable association. In the Sec11 ΔCTS system, the full-length LepCCt(14L) peptide showed markedly reduced contact frequency with Sec11 residues adjacent to the deleted CTS region (∼0.2), accompanied by a decreased contact frequency *via* the h-region of the peptide (Fig. S1*D*). These findings are consistent with the dissociation events observed during the simulations in (Fig. 6*C*). In contrast, the LepCCt(14L) carrying the shortened n-region retained h-region contacts with Sec11 residues upstream of the CTS deletion site, with contact frequencies reaching approximately 0.7 (Fig. S1*E*). These persistent interactions likely account for its continued association near the catalytic site despite removal of the Sec11 CTS.

## Discussion

Our experimental data, together with AlphaFold structural predictions, indicate that the Sec11 CTS forms a TM helix positioned within the TM window of the SPC, the site where signal sequences reside during cleavage. This structural arrangement suggested a potential role for the Sec11 CTS in substrate handling during signal peptide cleavage. To test this possibility, we examined which classes of signal sequences are efficiently processed and which are impaired in SPC lacking the Sec11 CTS.

Signal sequence hydrophobicity is a key determinant of protein targeting and translocation pathways in yeast (15, 20). Weakly hydrophobic signal sequences fail to engage the SRP and are instead targeted posttranslationally to the ER, where they require Sec62 to facilitate opening of the Sec61 translocon (25). We therefore considered whether the Sec11 CTS might preferentially contribute to the processing of signal sequences with specific hydrophobic properties. However, N-terminal signal sequences spanning a wide range of hydrophobicity were efficiently cleaved even in the absence of the Sec11 CTS. These results indicate that h region hydrophobicity is not the primary determinant of Sec11 CTS-dependent substrate selectivity.

**Figure 7:**
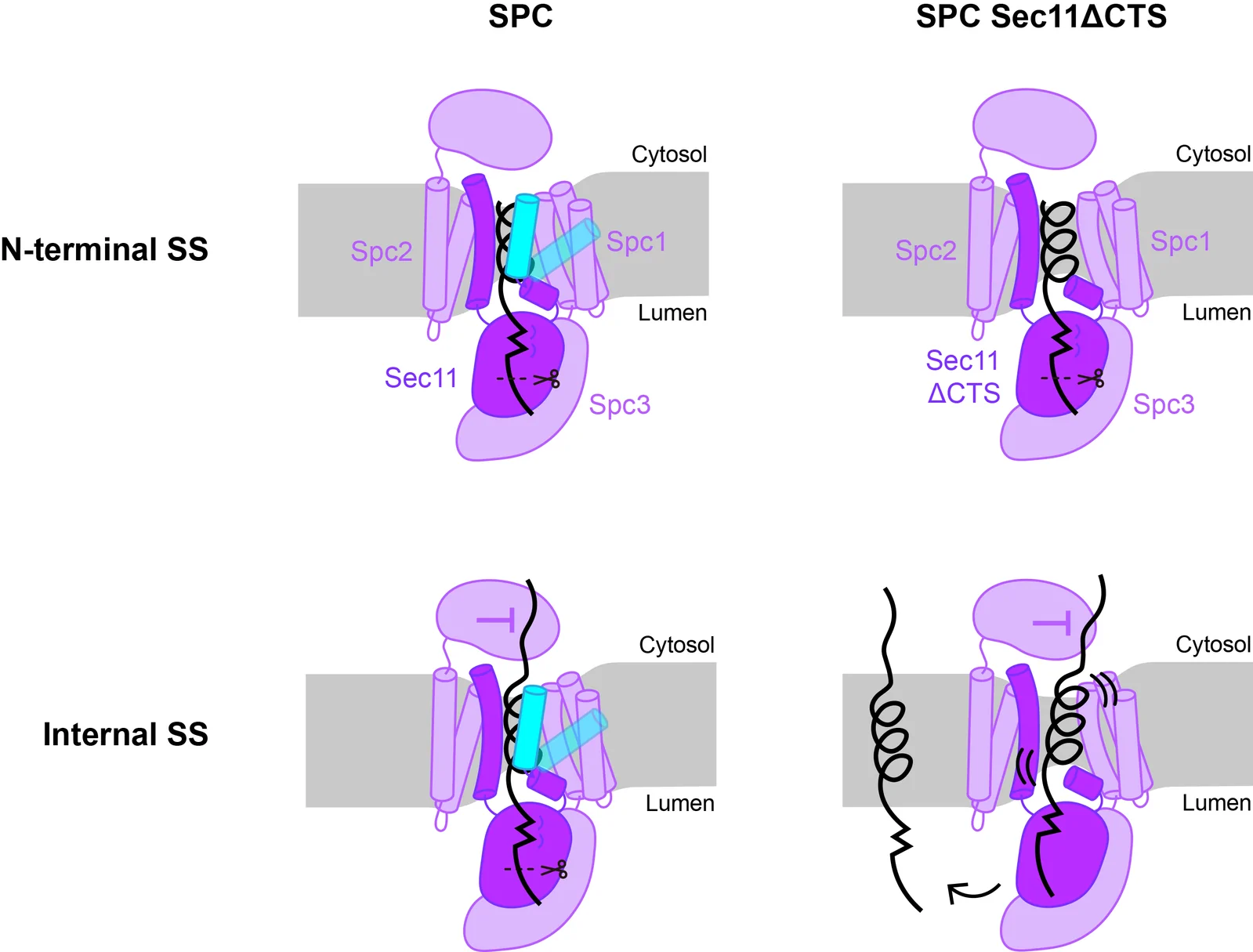
**Proposed models for function of the Sec11 CTS.** Schematic illustrations showing the potential roles and mechanisms of the Sec11 CTS based on the findings of this study and (9, 26). CTS, C-terminal short helix.

Next, we considered that the Sec11 CTS might function as a general gate regulating access to the TM window through conformational opening and closing within the membrane. This idea was motivated by the observation that the Sec11 CTS is unresolved in the cryo-EM structure of the human SPC, likely reflecting intrinsic flexibility (8). Supporting this view, the new cryo-EM structure of the human SPC with a bound signal peptide shows that the Sec11 CTS traverses the TM window and interacts with the signal peptide (26). These data suggest that the Sec11 CTS is flexible in the absence of substrate but, upon signal peptide binding, latches onto the substrate to secure it within the binding region (Fig. 7, left). If Sec11 CTS function as a latch is essential, its removal would be expected to broadly impair SPC activity and reduce cleavage across all classes of signal sequences. However, our results do not support this model. Cleavage of N-terminal signal sequences remains efficient in the absence of the Sec11 CTS, whereas processing of internal signal sequences is selectively compromised.

We previously showed that the cytoplasmic domain of Spc2 contributes to restricting the processing of internal signal sequences with extended n-regions (9). In the absence of the Sec11 CTS, such internal signal sequences may fail to be retained within the TM window of SPC due to the influence of the Spc2 cytoplasmic domain (Fig. 7). Consequently, the Sec11 CTS function may be particularly important for stabilizing internal signal sequences compared with N-terminal signal sequences.

MD simulations further provide insight into the structural consequences of Sec11 CTS removal. The overall membrane thickness at the TM window is largely unchanged in SPC lacking the Sec11 CTS compared with WT SPC (Fig. 6, *A* and *B*). Internal signal sequences appear capable of accessing the TM window independently of the Sec11 CTS. However, MD simulations reveal that substrates with extended n-regions can fail to be retained near the TM window in the absence of the Sec11 CTS, dissociating from the SPC. These observations suggest that the Sec11 CTS does not primarily modulate membrane properties but instead stabilizes internal signal sequences within the TM window, thereby facilitating their cleavage. Specifically, Sec11 CTS residues form a hydrophobic interface that stabilizes the h-region of LepCCt(14L), and this interaction becomes critical when the substrate carries an extended n-region.

Taken together, these data suggest that the Sec11 CTS functions as a substrate-stabilizing element that facilitates efficient processing of internal signal sequences, rather than as a general structural or gating component of the SPC.

## Experimental procedures

### Yeast strains and plasmids

The following *S. cerevisiae* strains were used in this study. W303-1α (*MATα*, *ade2*, *can1*, *his3*, *leu2*, *trp1*, *ura3*), *sec11*Δ (*MATα, sec11*Δ*::HIS3, ade2, can1, his3, leu2, trp1, ura3* [pRS415 *SEC11*]), *sec11*Δ*SPC3HA* (*MATα, sec11*Δ*::HIS3, SPC3HA::NAT, ade2, can1, his3, leu2, trp1, ura3* [pRS415 *SEC11*]), and *spc3-*4 (10).

The pRS424GPD-based plasmids encoding CPY signal sequence variants, LepCCt(14L, P + 1), Kar2, Rrt6, Ecm38, ppαF, and CPY were prepared in a previous study (18). Plasmids encoding pre-β-lactamase and pre-β-lactamase-2g pRS424GPD were constructed in pRS424GPD. SEC11 constructs—including WT *SEC11*, *sec11*ΔCTS, *SEC11-FLAG*, *sec11*ΔCTS-*FLAG*, *SEC11*–2g–3×HA, *sec11*ΔCTS–2g–3×HA, and *SEC11-GFP*—were cloned into pRS415 under control of the endogenous *SEC11* promoter. All constructs were prepared by site-directed mutagenesis or Gibson assembly.

### Plasmid shuffling

The *SEC11* ORF in W303-1α cells harboring pRS426GPD *SEC11* was replaced with *HIS3* amplified from pCgH plasmid (27) by homologous recombination, generating *sec11*Δ strain bearing pRS426GPD *SEC11* plasmid.

These cells were cotransformed with pRS415 plasmids expressing *SEC11*, *sec11*ΔCTS (Sec11 residues 148–167 were deleted), *SEC11-FLAG*, or *sec11*ΔCTS-*FLAG* under control of the endogenous promoter, plated on –Leu medium, and incubated at 30 °C for 2 days. Colonies were then replica-plated onto medium containing 5-FOA to select against the *URA3*-marked pRS426GPD-*SEC11* plasmid. Stable *sec11*Δ strains complemented with *SEC11* or mutant variants were obtained after at least three rounds of 5-FOA selection.

### Growth assay

Yeast cells were grown in selective medium at 24 °C to an absorbance (A)_600_ of 0.3 and 1.0. Cells (0.1 A_600_ units) were serially diluted, spotted onto –Leu plates, and incubated for 36 h at the indicated temperatures.

### Protein preparation and SDS-PAGE

Yeast cells were grown in selective medium at 24 to 30 °C to an A_600_ of 0.3 and 1.0. One A_600_ unit cells were harvested and stored at −20 °C until use. Frozen cell pellets were resuspended in 500 μl of distilled water, followed by addition of 75 μl of alkaline lysis buffer (1.85 N NaOH, 10 mM PMSF, 7.4% β-mercaptoethanol). Cell suspensions were vortexed vigorously and incubated on ice for 10 min. After cell lysis, proteins were precipitated by addition of 575 μl of 50% trichloroacetic acid and incubation on ice for 1 h. Protein precipitates were collected by centrifugation (20,238×*g*, 15 min, 4 °C), washed once with 500 μl of acetone, and centrifuged again (20,238×*g*, 15 min, 4 °C). Pellets were air-dried at room temperature and resuspended in 50 μl of SDS sample buffer with shaking at 30 °C. Samples were treated with Endo Hf (New England Biolabs) at 37 °C for 1 h prior to SDS-PAGE.

### Pulse labeling and immunoprecipitation

Pulse labeling and pulse chase experiments were performed as described previously (17, 28). Yeast cells were grown in selective medium at 24 to 30 °C to an A_600_ of 0.3 and 1.0. Cells (1.5 A_600_ units) were harvested by centrifugation (2170×*g*, 5 min, 4 °C), washed with methionine-free (−Met) medium lacking ammonium sulfate, and incubated at 30 °C for 10 min. Cells were then resuspended in 150 μl of −Met medium without ammonium sulfate, and labeled with [^35^S]Met (40 μCi per 1.5 A_600_ units of cells) for 5 min at 30 °C. After incubation, labeling was stopped by addition of 750 μl of ice-cold stop solution buffer (20 mM Tris–HCl, pH 7.5, 20 mM sodium azide). Cell pellets were collected by centrifugation (16,000×*g*, 1 min, 4 °C) and stored at −20 °C until use.

Radiolabeled cell pellets were resuspended in 100 μl of lysis buffer (20 mM Tris–HCl, pH 7.5, 1% SDS, 1 mM DTT, 1 mM PMSF, and Protease Inhibitor Cocktail) and mixed with 100 μl of cold glass beads. Cell suspensions were disrupted by vortexing twice for 2 min, with 1-min intervals on ice, incubated at 60 °C for 15 min, and centrifuged (6000 *g*, 1 min, 4 °C). Supernatants were mixed with 500 μl of immunoprecipitation buffer (15 mM Tris–HCl, pH 7.5, 0.1% SDS, 1% Triton X-100, and 150 mM NaCl), 1 μl of anti-HA antibody, and 20 μl of prewashed protein G-agarose beads (Thermo Scientific Pierce) and incubated for 3 h at room temperature with rotation.

Beads were washed twice with immunoprecipitation buffer, once with ConA buffer (500 mM NaCl, 20 mM Tris–HCl, pH 7.5, and 1% Triton X-100), and once with buffer C (50 mM NaCl and 10 mM Tris–HCl, pH 7.5). Bound proteins were eluted with 50 μl of SDS sample buffer (50 mM DTT, 50 mM Tris–HCl, pH 7.6, 5% SDS, 5% glycerol, 50 mM EDTA, 1 mM PMSF, Protease Inhibitor Cocktail (Quartett), and bromophenol blue) by incubation at 60 °C for 15 min.

### Coimmunoprecipitation

Yeast cells were grown overnight in selective medium at 24 °C to an A_600_ of 0.3 and 1.0. Cells (20 A_600_ units) were harvested by centrifugation and resuspended in 400 μl of Hepes buffer (50 mM Hepes-KOH, pH 6.8, 150 mM KOAc, 2 mM Mg(OAc)_2_, 1 mM CaCl_2_, 15% glycerol) supplemented with 0.1% Triton X-100, protease inhibitor cocktail (Quartett) and 2 mM PMSF. Cells were lysed by bead-beating (five cycles of 1 min vortexing at maximum speed with 2-min intervals on ice). Cell lysates were clarified by sequential centrifugation at 6500 rpm for 10 min and 10,000 rpm for 20 min at 4 °C.

Clarified lysates (300 μl) were adjusted to a final concentration of 1% Triton X-100 by addition of an equal volume of Hepes buffer containing 1.9% Triton X-100 and rotated for 1 h at 4 °C. Insoluble material was removed by centrifugation at maximum speed for 30 min at 4 °C. A total of 550 μl supernatant was transferred to a new tube, and 27.5 μl (5%) was saved as input. Lysates were precleared with 25 μl protein G–agarose beads (Pierce) for 1 h at 4 °C. After removal of beads by centrifugation, 500 μl of precleared lysate was incubated with 25 μl protein G–agarose and 2 μl mouse anti-HA antibody (BioLegend) overnight at 4 °C with rotation. Beads were washed three times with 1 ml of Hepes buffer containing 1% Triton X-100. Bound proteins and input samples were mixed with 5x SDS sample buffer, incubated at 60 °C for 15 min, treated with Endo Hf at 37 °C for 1 h, and resolved by SDS-PAGE.

### Quantification and statistics

Radiolabeled proteins were detected using a Typhoon FLA 9500 phosphoimager. Band intensities were quantified using MultiGauge version 3.0 software (Fujifilm). Cleavage efficiency was calculated as cleavage (%) = cleaved (mature) products × 100/cleaved (mature) + full-length (precursor) products. Cell growth assays were imaged using a ChemiDoc XRS+ system.

Statistical analyses were performed using MATLAB (MathWorks, Inc.).

### Yeast SPC structure predictions

The structure of the yeast SPC was predicted using the AlphaFold server prediction (https://alphafoldserver.com/) (29) and validated in our previous study (9). The WT SPC model was generated using the full-length sequences of the yeast SPC subunits. The SPC lacking the Sec11 CTS was generated by removing the C-terminal 20 amino acids (KYAKFALLGMLGLSALLGGE) of the Sec11 sequence.

The structure of the LepCCt(14L) peptide with and without the n-region was also predicted using the AlphaFold server (29). The LepCCt(14L) peptide with n-region was generated using the full-length sequence of the LepCCt(14L) peptide, while the LepCCt(14L) peptide without n-region was generated by removing the first 40 amino acids (MCVDKFFFAPKRRERQAAAQAAAGDSLDKATLKKVAPKPG) of the LepCCt(14L) sequence (Fig. 4*A*). The SPC models with the LepCCt(14L) peptide were generated using the AlphaFold multimer option, where the SPC subunits and the LepCCt(14L) peptide were input as separate chains.

### MD simulations system setup

The predicted structures of the yeast SPC with and without the Sec11 CTS were CG using the latest Martinize release (30). An elastic network was applied to each protein subunit with a force constant of 1300 kJ mol^−1^ nm^−2^ and a cutoff of 0.8 nm. Additionally, intermolecular harmonic bonds were added between certain residues of the SPC subunits as described in (9) to maintain the stability of the SPC during the simulations.

The SPC models with the LepCCt(14L) peptide were also CG using the Martinize tool (30), with the same elastic network parameters as described above. The full-length LepCCt(14L) peptide used in the simulations comprises the sequence MCVDKFFFAPKRRERQAAAQAAAGDSLDKATLKKVAPKPGLIKKKKLLLLLLLLLLLLLLVPSAQAAYEPFQ (n-region, h-region in bold, and c-region), while the truncated peptide contains only LIKKKKLLLLLLLLLLLLLLVPSAQAAYEPFQ (shortened n-region, h-region in bold, and c-region). The peptide was treated as a separate molecule in the coarse-graining process, and no additional harmonic bonds were applied between the peptide and the SPC subunits.

The WT SPC and SPC lacking the Sec11 CTS, both with and without the LepCCt(14L) peptides, were embedded in a complex lipid bilayer that mimics the ER membrane composition (Monje–Galvan and Klauda, 2015) using INSANE (31). The lipid bilayer consisted of a mixture of phosphatidylcholines DYPC (16:1–16:1) and YOPC (18:1–16:1), phosphatidylinositols POPI (18:1–16:0) and PYPI (16:1–16:0), phosphatidylethanolamines DYPE(16:1–16:1) and YOPE (18:1–16:1), phosphatidic acid YOPA (18:1–16:1), phosphatidylserine YOPS (18:1–16:1) and POPS (18:1–16:0), and ergosterol symmetrically distributed between the upper and lower leaflets, and was solvated with Martini water and 0.15 M NaCl.

The simulation systems were energy minimized for 10,000 steps using the steepest descent algorithm, followed by five steps of equilibration using time steps of 10, 15, and 20 fs. The equilibration runs were performed at 310 K and 1 bar pressure. The temperature was maintained using the Berendsen thermostat, and the pressure coupling was performed using the Berendsen barostat (32). The semi-isotropic pressure coupling was applied with a compressibility of 3 × 10^−4^ bar^−1^ and a time constant of 1 ps were applied separately to the protein, lipids, and solvent.

The production runs were performed in four repeats for 30 μs each, with a time step of 20 fs, at 310 K and 1 bar using the V-rescale thermostat (33) and Parrinello–Rahman barostat (34), respectively. All simulations were performed using GROMACS 2025.2 (35) with a time step of 20 fs.

The simulations with the LepCCt(14L) peptide were performed with the same parameters as described above, using three repeats for each system, with a production run of 15 μs each.

### Membrane thickness

Membrane thickness computed for a system composed of the yeast SPC embedded into a POPC (1-palmitoyl-2-oleoyl-sn-glycerol-3-phophocholine) membrane at atomistic or Martini 3 resolution. The membrane thickness at the TM window of SPC was calculated as the distance between the position of the phosphate beads (Martini PO4 beads) in the upper and lower leaflets of the lipid bilayer. For this, the trajectory frames from 5 to 15 μs of the production runs were extracted and analyzed with the *gmx density* tool in GROMACS (35). The density profiles of the PO4 beads were calculated along the *z*-axis, and the absolute difference between the lipids only (control) and the SPC-containing simulations was calculated.

### Distances between peptides and the protein

The distance between the LepCCt(14L) peptide and the SPC was calculated as the distance between the ALA backbone bead of the peptide and the HIS83 backbone bead of the Sec11, which is one of the residues of the catalytic region. The distance was calculated using *the gmx distance* tool in GROMACS (35).

### Contact frequency

Contact frequency was calculated as a pairwise metric between the LepCC(14L) with short or extended n-region residues and WT Sec11 or ΔCTS. For each frame from 5 to 15 μs trajectory, residue pairs from two groups were identified as being in contact if the distance between any pair of atoms fell below a specified cut-off distance (0.8 nm) using MDAnalysis (36). Each contact was incremented by 1 for the corresponding residue pair. Contact counts were normalized by dividing by the total number of frames, yielding contact frequency values ranging from 0 (no contact throughout the analyzed trajectory) to 1 (contact maintained in every frame). Average contact frequency maps were generated by computing the mean across all replicas.

### Molecular visualization

MD simulation snapshots were visualized using VMD (37).

## Data availability

All the data are included in the article. Original scans of the gel data are available upon request.

## Supporting information

This article contains supporting information.

## Conflict of interest

The authors declare that they have no conflicts of interest with the contents of this article.
