# Supplementary material for: The Sec11 C-terminal short helix promotes productive engagement of internal signal sequences with the signal peptidase complex
Source: J Biol Chem. 2026 Aug 12;302(10):113442. doi: 10.1016/j.jbc.2026.113442 (PMC13578380; doi:10.1016/j.jbc.2026.113442)
Supplement: Supplementary file 1 [file mmc1.docx]

**Supporting Information**

**The Sec11 C-terminal short helix promotes productive engagement of internal signal sequences with the signal peptidase complex**

Yeonji Chung^1^, Mariia Borbuliak^2,3^, Sanghun Hwang^1^, Paulo C. T. Souza^2,3^ and Hyun Kim^1,*^

^1^ School of Biological Sciences and Institute of Biodiversity, Seoul National University, Seoul 08826, South Korea

^2^ Laboratoire de Biologie et Modélisation de la Cellule, UMR 5086, CNRS, Ecole Normale Supérieure de Lyon, 46 Allée d’Italie, 69364, Lyon, France

^3^ Centre Blaise Pascal de Simulation et de Modélisation Numérique, Ecole Normale Supérieure de Lyon, 46 Allée d’Italie, 69364, Lyon, France

^*^ To whom correspondence should be addressed: Hyun Kim, School of Biological Sciences and Institute of Microbiology, Building 504-421, Seoul National University, Seoul 08826, South Korea; Phone: Int+82-2-880-4440; Fax: Int+82-2-872-1993; Email**:**  [joy@snu.ac.kr](mailto:joy@snu.ac.kr)

List:

Figures S1A-E are included.

**Figure S1.** Average pairwise residue–residue contact frequencies from MD simulations of SPC containing Sec11 WT or Sec11 ΔCTS in complex with LepCCt(14L) peptides with or without the n-region (+n-region or -n-region). (A) 3D surface representation of average contact frequency between LepCCt(14L) and Sec11 WT residues. Contact frequency is color-coded from white (low) to orange/red (high). Other SPC subunits are shown as transparent surfaces. (B–E) Corresponding 2D residue–residue contact heatmaps: (B) Sec11 WT + LepCCt(14L), (C) Sec11 WT + LepCCt(14L) lacking the n-region, (D) Sec11 ΔCTS + LepCCt(14L), and (E) Sec11 ΔCTS + LepCCt(14L) lacking the n-region. Contacts were defined using a 0.8 nm distance cutoff. In all heatmaps, darker colors indicate higher contact frequency.
